# The Relationships of Specific Cognitive Control Abilities with Objective and Subjective Sleep Parameters in Mild Cognitive Impairment: Revealing the Association between Cognitive Planning and Sleep Duration

**DOI:** 10.3390/brainsci14080813

**Published:** 2024-08-14

**Authors:** Areti Batzikosta, Despina Moraitou, Paschalis Steiropoulos, Georgia Papantoniou, Georgios A. Kougioumtzis, Ioanna-Giannoula Katsouri, Maria Sofologi, Magda Tsolaki

**Affiliations:** 1 Laboratory of Psychology, Department of Cognition, Brain and Behavior, School of Psychology, Faculty of Philosophy, Aristotle University of Thessaloniki (AUTh), 54124 Thessaloniki, Greece; demorait@psy.auth.gr; 2 Laboratory of Neurodegenerative Diseases, Center of Interdisciplinary Research and Innovation (CIRI-AUTH), Balcan Center, Buildings A & B, 57001 Thessaloniki, Greece; gpapanto@uoi.gr (G.P.); tsolakim1@gmail.com (M.T.); 3 Department of Respiratory Medicine, Medical School, Democritus University of Thrace, 68100 Alexandroupolis, Greece; steiropoulos@yahoo.com; 4 Laboratory of Psychology, Department of Early Childhood Education, School of Education, University of Ioannina, 45110 Ioannina, Greece; m.sofologi@uoi.gr; 5 Institute of Humanities and Social Sciences, University Research Centre of Ioannina (URCI), 45110 Ioannina, Greece; 6 Department of Turkish Studies and Modern Asian Studies, Faculty of Economic and Political Sciences, National and Kapodistrian University of Athens, 15772 Athens, Greece; gkougioum@ppp.uoa.gr; 7 Department of Psychology, School of Health Sciences, Neapolis University Pafos, Pafos 8042, Cyprus; 8 Department of Occupational Therapy, Faculty of Health and Caring Sciences, University of West Attica, 12243 Athens, Greece; ykatsouri@uniwa.gr; 9 Greek Association of Alzheimer’s Disease and Related Disorders (GAADRD), Petrou Sindika 13 Str., 54643 Thessaloniki, Greece

**Keywords:** cognitive abilities, executive functions, mild cognitive impairment, sleep, sleep disturbances

## Abstract

This study aimed to examine the associations between specific sleep parameters and specific aspects of cognitive functioning in individuals diagnosed with mild cognitive impairment (MCI), compared with healthy controls (HCs) by using cognitive, subjective, and objective sleep measures. A total of 179 participants were enrolled, all aged ≥ 65 years (mean age = 70.23; SD = 4.74) and with a minimum of six years of education (mean = 12.35; SD = 3.22). The sample included 46 HCs (36 females), 75 individuals with amnestic MCI (aMCI) (51 females), and 58 individuals with non-amnestic MCI (naMCI) (39 females). Inhibition, cognitive flexibility as a combined application of inhibitory control and set shifting or task/rule switching, and planning were examined. The following D-KEFS subtests were administered for their evaluation: Verbal Fluency Test, Color–Word Interference Test, and Tower Test. Self-reported sleep questionnaires (Athens Insomnia Scale, Stop-Bang questionnaire, and Pittsburg Sleep Quality Index) were used for subjective sleep assessments. Actigraphy was used for objective sleep measurements. Mixed-measures ANOVA, MANOVA, and one-way ANOVA, as well as the Scheffe post hoc test, were applied to the data. The results showed that the three groups exhibited statistically significant differences in the Tower Test (total achievement score, total number of administered problems, and total rule violations). As regards objective sleep measurements, the total sleep time (TST) was measured using actigraphy, and indicated that there are significant differences, with the HC group having a significantly higher mean TST compared to the naMCI group. The relationships evaluated in the TST Tower Test were found to be statistically significant. The findings are discussed in the context of potential parameters that can support the connection between sleep duration, measured as TST, and cognitive planning, as measured using the Tower Test.

## 1. Introduction

Mild cognitive impairment (MCI) has emerged as a condition that precedes the full manifestation of Alzheimer’s disease, serving as a ’situation’ characterized by milder cognitive disturbances and everyday functioning maintenance [1]. MCI can be categorized into different subtypes based on the nature of cognitive impairments observed. Two common subtypes are amnestic MCI (aMCI) and non-amnestic MCI (naMCI), based on whether the main cognitive impairments are in memory or (an)other cognitive domain(s), respectively [1,2]. Subtyping helps in understanding the specific cognitive domains affected, and may provide insights into the likelihood of progression to specific types of more severe cognitive disorders. Beyond its impact on daily functioning, MCI appears to exert a negative influence on a crucial pillar of our health—sleep.

Mechanisms associated with the onset of this condition have been considered to worsen the quality and duration of sleep, affecting an individual’s ability to achieve deep, restorative sleep [3]. Sleep disturbances can negatively impact cognitive control, commonly known as executive functions, which involve a range of higher-level cognitive processes that direct goal-oriented actions [4,5]. Disruptions in cognitive control can significantly undermine cognitive performance and exacerbate the risk of cognitive decline and neurodegenerative conditions over time. Consequently, ensuring the maintenance of optimal cognitive control emerges as a critical imperative for safeguarding comprehensive cognitive health and wellbeing, warranting further investigation and attention in both clinical and research settings. 

The mechanisms associated with MCI, which could impact both sleep and cognitive functions, are varied and intricate. MCI has the potential to induce disruption in the neural networks responsible for sleep regulation, like the wakefulness–sleep system. This disruption may result in instability throughout the sleep cycle and diminished brain rejuvenation [6]. Irregularities in the reassignment of neurotransmitters, such as norepinephrine and dopamine associated with MCI, can impact the equilibrium between wakefulness and sleep, potentially causing disruptions in both sleep patterns and cognitive functions [7]. Brain inflammation could potentially contribute to MCI by influencing the function of neurons and glial cells, thereby affecting both sleep and cognitive functions [8]. Additionally, the loss of connections between neurons and the disappearance of nerve cells can contribute to MCI and have implications for cognitive functions [9]. Furthermore, studies have shown that patients with MCI may have decreased volume in the amygdala, an area associated with the regulation of sleep [10,11,12]. Ultimately, MCI has the potential to expedite neurodegenerative processes linked to Alzheimer’s disease, affecting both sleep and the main functions of cognitive control [7].

These mechanisms probably collaborate with one another, along with other as-yet-unidentified factors, to instigate disturbances in sleep and cognitive functions related to MCI. An in-depth understanding of the intricate relationships between specific sleep parameters and specific cognitive functions is essential, particularly in the context of MCI. As individuals with MCI experience a decline in cognitive abilities, exploring the interrelationships between cognition and sleep quality and quantity becomes crucial for comprehending the factors potentially influencing the progression of cognitive change in this population. 

### 1.1. Aims and Hypotheses of the Present Study

The study aimed to reveal the associations between specific sleep parameters and specific dimensions of cognitive control functioning in two MCI subtypes to highlight their interrelations in the early stages of cognitive impairment.

### 1.2. Based on the Theoretical Framework, the Following Hypotheses Were Formulated

With respect to sleep disturbances, we hypothesized that individuals with aMCI and naMCI will have more disturbed sleep than cognitively healthy (HC) older adults, and would differ between each other at least in specific sleep parameters **(Hypotheses 1a and 1b, respectively)**. Neurobiological changes associated with both aMCI and naMCI can contribute to disruptions in sleep regulation. In MCI, ongoing neurodegenerative processes may impact brain areas associated with sleep–wake regulation, including the hypothalamus and brainstem nuclei. These alterations can lead to disturbances in the normal sleep–wake cycle, leading to difficulties in initiating sleep, maintaining sleep continuity, or achieving restful sleep. On the other hand, cognitive impairment, a hallmark of MCI regardless of subtype, could exacerbate sleep disturbances. Increased cognitive arousal during the night, possibly due to ongoing neural dysfunction, may contribute to sleep fragmentation and poorer sleep quality in individuals with MCI. Additionally, cognitive decline may impair the ability to self-monitor and regulate sleep behaviors, further exacerbating potential sleep disturbances. Individuals with aMCI are expected to show a greater percentage reduction in total sleep time (TST) and more fragmented sleep, manifested by longer durations of nighttime awakenings. This suggests that memory-related neural deficits in aMCI may particularly affect the continuity of sleep [9]. In contrast, patients with naMCI may exhibit increased sleep fragmentation, evidenced by frequent brief awakenings, and greater variability in sleep onset and wake times. These differences can be attributed to the distinct cognitive and neurobiological profiles in naMCI, which may affect the regulation of sleep–wake cycles [7].

We also hypothesize that performance in specific executive functioning tests will be associated with specific sleep parameters (Hypothesis 2). The multifaceted relationship between executive functioning and sleep underscores the complex interplay between these processes. Disruptions in sleep duration can significantly relate to cognitive control performance in terms of specific abilities. Understanding this relationship has implications for both basic neuroscience research and clinical interventions aimed at optimizing cognitive health.

**Hypothesis** **2a.** *Hence, longer total sleep time (TST) will correlate with higher performance in tasks requiring at least a combination of cognitive control abilities. This prediction is based on the literature according to which sleep plays a crucial role in supporting cognitive control processes essential for long-term planning* [13].

**Hypothesis** **2b.** *Improved sleep quality, as indicated by measures like decreased wake after sleep onset (WASO) and increased sleep efficiency (SE), will be associated with enhanced performance in tasks involving inhibitory control* [14].

## 2. Materials and Methods

### 2.1. Design

This study represents the first cross-sectional phase of a broader project, comparing three groups—(a) cognitively healthy older adults (HC ≥ 65 years), (b) people with aMCI, and (c) people with naMCI—in specific sleep parameters, specific cognitive control abilities, and their relationships.

### 2.2. Participants

Regarding the size of the sample, a power analysis was performed using G*Power [15]. The analysis indicated that a minimum of 148 participants would be required to achieve a power level of 0.80. A total of 185 individuals were recruited and assessed for their cognitive health. Among them, one individual was classified as having Subjective Cognitive Impairment (SCI), another with early dementia, and four others were receiving medication for sleep disorders. Consequently, these six participants were excluded from this study. From the remaining 179 participants, 46 were found to be cognitively healthy, 75 participants were diagnosed with aMCI, and 58 participants were diagnosed with naMCI. Every participant gave their consent to participate in this study. 

Hence, the study included 179 participants, 53 men and 126 women, with a mean age of 70.23 (SD = 4.74) years and a mean educational level (measured as years of schooling) of 12.35 (SD = 3.22) years. Eligibility for this study required individuals to be older than 65 and to have completed a minimum of six years of education. To classify participants into categories of healthy cognitive status, aMCI, or naMCI, all individuals underwent a detailed neuropsychological assessment based on Petersen’s diagnostic criteria [2] and *Diagnostic and Statistical Manual of Mental Disorders, Fifth Edition, Text Revision* (DSM-5-TR) [16]. 

### 2.3. Inclusion Criteria

The control group consisted of 46 healthy community-dwelling adult volunteers with excellent cognitive health. Their MoCA score ranged from 27 to 30 (M = 27.8, SD = 0.74). 

The aMCI group consisted of 75 adults diagnosed with MCI during the last two years. The inclusion criteria were based on the DSM-5-TR criteria for Mild Neurocognitive Disorders [16]. Their diagnosis, in addition to the extended neuropsychological assessment, was supported by neurological examination, neuroimaging, and psychiatric assessment. The inclusion criteria were as follows: (a) diagnosis of Minor Neurocognitive Disorder, and (b) 1.5 standard deviations (SDs) below the normal mean in at least the episodic memory test. According to the evaluations, 75 MCI participants were diagnosed with the amnestic type. Their MoCA test ranged from 24 to 29 (M = 24.59, S.D. = 2.7). 

The naMCI group consisted of 58 adults diagnosed with MCI during the last two years. The inclusion criteria were as follows: (a) diagnosis of Minor Neurocognitive Disorder, and (b) 1.5 standard deviations (SDs) below the normal mean in at least one cognitive domain, other than memory, according to the neuropsychological tests. According to the evaluations, 58 MCI participants were diagnosed with the non-amnestic type. Their MoCA test ranged from 20 to 29 (M = 26.10, S.D. = 2.47). 

### 2.4. Exclusion Criteria

Participants were excluded from the three groups based on the following criteria: (a) psychiatric disorders in their history; (b) substance abuse or alcoholism; (c) a past traumatic brain injury; (d) neurological disorders; (e) sleep disorders; (f) medication for sleep disorders; (g) medication for depression or anxiety; and for the HC group specifically, the presence of cognitive complaints. 

All participants underwent a thorough neuropsychological evaluation for diagnostic purposes. This assessment was conducted at the Greek Association of Alzheimer’s Disease and Related Disorders and included the following tools: the Geriatric Depression Scale [17,18], the Beck Depression Inventory [19], the Beck Anxiety Inventory [20], and the Short Anxiety Screening [21,22] to screen for affective disorders. Additionally, the Neuropsychiatric Inventory [23,24] was used to identify neuropsychiatric symptoms. The Mini-Mental State Examination [25,26] and the Montreal Cognitive Assessment [27,28] were employed to evaluate global cognitive status, while the Functional Cognitive Assessment [29] assessed executive functions through six daily activities. Further standardized cognitive tests were administered to evaluate memory, attention, executive functions, and language skills. The Global Deterioration Scale (GDS) [30] was utilized to gauge the progression of cognitive decline, with stage 1 indicating no cognitive decline and normal functioning, and stage 3 indicating mild cognitive impairment (MCI). For a detailed overview of all the neuropsychological tests utilized, refer to Tsolaki et al. [31].

No significant differences between the three groups were found in terms of age, F (2, 176) = 2.977, *p* = 0.054; years of schooling, F (2, 176) = 0.452, *p* = 0.637; and gender, χ^2^ = 1.849, *p* = 0.397 (see Table 1).

### 2.5. Ethics

Participants were informed about the study’s aims through oral and written communication and were guaranteed that their data would remain confidential. They signed a written consent form, indicating their voluntary participation and their ability to withdraw from the study whenever they chose. The collection of demographic data, including age, gender, and education, was conducted in line with European Union legislation effective 28 May 2018, which authorizes the use of sensitive personal information for research purposes. Participants were notified and consented to the removal of their data from the web database upon written request. The research protocol was approved by the Scientific and Ethics Committee of the Greek Association of Alzheimer’s Disease and Related Disorders (Approval Code: 29/15-02-2017) and complied with the Declaration of Helsinki guidelines. 

### 2.6. Procedure 

Undergraduate psychology interns assisted in recruiting participants from the Daycare Centers of the Greek Association of Alzheimer’s Disease and Related Disorders and the Aristotle University of Thessaloniki. Volunteers who met the inclusion criteria were invited to participate and, if they agreed, were contacted by a study psychologist who provided detailed information about the study’s purpose and procedures. Participants then scheduled two morning appointments within a week, each lasting up to an hour, to complete the tests. Written informed consent was obtained at the first appointment, ensuring confidentiality and explaining the study’s objectives. The tests were administered individually in a quiet, comfortable environment, using two different versions of the testing battery to avoid order effects. No compensation was provided to participants for their involvement.

### 2.7. Instruments

#### 2.7.1. Cognitive Measures

Referencing literature that identifies executive function impairments in MCI patients, we chose to assess executive functions or cognitive control abilities, namely inhibitory control, set-shifting, cognitive flexibility, and planning, with the use of specific tests of the Delis–Kaplan Executive Function System (D-KEFS) [32]. Other cognitive dimensions such as memory capacity and theory of mind were also measured in detail, but in this article, we will limit ourselves to presenting the results for the cognitive control abilities.

#### 2.7.2. D-KEFS Verbal Fluency Test, Standard Form–D-KEFS VF, SF 

The D-KEFS Verbal Fluency Test assesses crystallized intelligence and executive functions through the following three conditions: Phonemic fluency, Semantic fluency, and Category Switching. Each condition tests different cognitive abilities, such as generating words based on specific phonemes, producing words within a semantic category, and switching between semantic categories. Scoring is based on correct responses, with each accurate word and correct category switch earning one point [32]. 

#### 2.7.3. D-KEFS Color–Word Interference Test, Standard Form–D-KEFS C-WIT, SF

This test assesses the capacity to inhibit automatic responses and involves the following four conditions: naming basic colors, reading color words, naming the ink color of incongruent color words (inhibition), and switching between naming colors and reading words (inhibition/switching). The test, inspired by the Stroop effect, includes time limits of 90 s for the first two conditions, and 180 s for the latter two [32]. The scoring involves measuring completion time and categorizing errors across four conditions, with specific attention being paid to uncorrected and self-corrected errors, allowing for the evaluation of processing speed, inhibitory control, and cognitive flexibility. 

#### 2.7.4. D-KEFS Tower Test, Standard Form–D-KEFS–TT, SF

In the context of this test, participants are tasked through the construction of towers with five disks across three vertical pegs, aiming to solve each problem using the minimum number of moves possible [32]. The setup includes a wooden board with three pegs, five wooden disks of varying size and shades of blue, a timer, a recording tool, and a stimulus booklet. The test encompasses nine problems of increasing difficulty, each assigned a specific time for completion, as follows: 30 s for the first three problems, 60 s for the fourth, 120 s for the fifth and sixth, 180 s for the seventh, and 240 s for the eighth and ninth problems. Two rules are communicated both orally and in writing—only one disk can be moved at a time, and no disk should be placed on top of a smaller one. The examiner arranges the disks on the pegs in a specific order, presenting the participant with a picture of the tower’s final desired configuration. The participant starts with two disks in the first problem, and the number increases during the test. If a participant struggles, the examiner provides the solution for the first and second problems. The scores derived from the D-KEFS–TT in this study include (a) the total number of administered problems, (b) the total number of rule violations, and (c) the total achievement score based on correct solutions (scoring according to the number of moves).

The D-KEFS Tower Test is a tool designed to assess complex executive functions, with factor analysis showing positive loadings on skills such as spatial planning, rule learning, inhibition, and the ability to establish and maintain cognitive sets. Its validity has been confirmed through studies involving individuals with MCI, with recent research revealing that people with MCI tend to score lower on the test compared to a control group [33,34].

### 2.8. Sleep Measures

Subjective measures: self-reported questionnaires.

#### 2.8.1. Athens Insomnia Scale

The Athens Insomnia Scale (AIS) includes eight items in a self-assessment format to evaluate insomnia severity in adults, covering aspects like sleep onset, maintenance, and daytime consequences. Each item is rated on a scale from 0 to 3, with higher scores indicating more severe insomnia. The AIS has been validated for use in the Greek population, showing a high sensitivity (93%) and specificity (85%) for diagnosing insomnia, with a total score of 6 or higher being the optimal cutoff for identifying cases [35]. 

#### 2.8.2. Stop-Bang Questionnaire

The Stop-Bang questionnaire is an eight-question screening tool used to evaluate the risk of obstructive sleep apnea (OSA) [36]. It assesses factors such as snoring, tiredness, observed apneas, high blood pressure, body mass index (BMI), age, neck circumference, and gender. Each “yes” response scores one point, with a total possible score of 8, where higher scores indicate a greater likelihood of OSA. The tool has been validated in the Greek population, demonstrating that a score of 4 or more effectively identifies individuals with OSA [37].

#### 2.8.3. The Pittsburgh Sleep Quality Index (PSQI)

The Pittsburgh Sleep Quality Index (PSQI) is a 19-item self-report tool designed to evaluate sleep quality over the past month, encompassing seven areas including sleep latency, duration, and disturbances [38]. Scores range from 0 to 21, with higher scores reflecting a worse sleep quality. The Greek version of the PSQI has been validated and is considered highly reliable and effective for both clinical and research purposes, including among individuals with sleep disorders and cancer patients [39,40,41]. 

#### 2.8.4. Sleep Diary

A sleep diary is a self-reported log that tracks an individual’s sleep and wake times, along with other relevant details. It serves as a useful tool for diagnosing and managing circadian rhythm sleep disorders and is often used alongside actigraphy. In our study, participants kept a sleep diary for one week while also wearing an actigraphy device. The diary recorded various details, including intended and actual wake times, time out of bed, daytime naps and exercise, medication, sleep aids, caffeine, and alcohol intake [42].

#### 2.8.5. Objective Sleep Measures

##### Actigraphy

Actigraphy, a method that infers sleep–wake patterns by analyzing movement data collected through an actigraph, is increasingly utilized in research due to its capacity to monitor activity in natural environments [43]. Participants in all groups were provided with an actigraphy device for seven days. This wrist-worn device, resembling a regular watch, records data that when decoded, offer insights into the participant’s sleep–wake cycle. We used Philips Respironics Actiware, specifically the Actiwatch Spectrum Pro, version 5.57.0006. 

**Figure 1:**
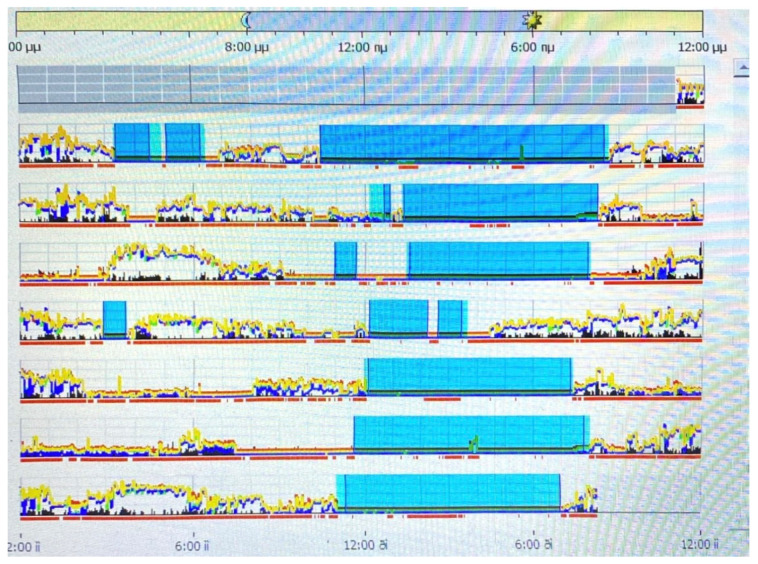
A graphical representation of a participant’s phases of activity and rest over the course of a week.

The Actiwatch captures motion and light data, offering insights into general activity levels, sleep patterns, wake and nap times, as well as details regarding sleep quantity and quality. Data collected by actigraphy devices serve various purposes in sleep medicine, such as investigating sleep disorders, circadian rhythm disturbances, and daytime activity levels. These data can be analyzed with a focus on sleep patterns or the complete circadian cycle. Sleep analysis can measure the start and end times of sleep, total sleep duration, sleep onset latency, the duration of nighttime awakenings, and overall sleep efficiency. (see Figure 1). The memory chip can be read using a basic device, and the data can be visualized with a histogram on a regular computer, laptop, or tablet. Parameters like time in bed, total sleep time, sleep latency, the number and duration of awakenings, and daytime sleep intervals are automatically calculated and displayed.

### 2.9. Statistical Analysis 

Statistical analysis was performed using IBM SPSS Statistics, Version 27 [44]. To test whether the three groups differed in cognitive performance, as well as in sleep measures, the following analyses were conducted: (a) multivariate analysis of variance (MANOVA) and (b) one-way ANOVA. Τo control for multiple testing, a Bonferroni correction was applied. A *p*-value < 0.005 was considered indicative of statistical significance for the cognitive control performance (i.e., significant *p* = 0.05/9 (dependent variables) = 0.005) and a *p*-value < 0.008 was considered indicative of statistical significance for the subjective and objective sleep measures (i.e., significant *p* = 0.05/6 (dependent variables) = 0.008). The effect size was estimated using η^2^, and post hoc comparisons were conducted using the Scheffe test. Pearson correlation coefficients were computed to examine the linear relationships between the specific variables of sleep and cognitive control, which were found to be significantly affected by diagnosis [total sleep time and each D-KEFS–TT, SF variable (total number of problems given, total number of violations, and total achievement score)]. Mediation analysis within the context of Structural Equation Modeling (path models with mediators) was also conducted using JASP 16 [45] to examine whether the diagnostic group (the predictor) directly or/and indirectly—via a sleep parameter—affects performance in cognitive control abilities. We considered “diagnosis” as the predictor variable and the total sleep time as the potential mediator. D-KEFS–TT, SF test scores (including the total number of problems given, total number of violations, and total achievement score) were set as outcome variables. 

## 3. Results 

### 3.1. Cognitive Control Performance

The sum of correct responses of each participant on each subtest of the D-KEFS VF and the D-KEFS C-WIT test was calculated, and similarly, the scores for the D-KEFS–TT, SF were computed. MANOVA was conducted to investigate the differences in cognitive control performance between the three groups on the above tests and subtests. As dependent variables, four cognitive scores of the D-KEFS VF were identified [1. Phonemic fluency (total *n* of correct words), 2. Semantic fluency (total *n* of correct words), 3a. Semantic fluency with switching (total *n* of correct words), 3b. Semantic fluency with switching (total *n* of switches)], two from the D-KEFS C-WIT test (condition iii (correct inhibition scores) and condition iv (correct inhibition/switching scores)), and three for the D-KEFS–TT, SF test (total number of problems given, the total number of violations, and the total achievement score)]. The diagnostic group was identified as the independent variable.

The results indicate that the diagnostic group had a significant effect (V = 0.960, F (18, 338) = 17.319, *p* < 0.001, η^2^ = 0.48) on the dependent variables. In particular, the diagnostic group effect was significant only for the D-KEFS–TT, SF test on (a) the total number of problems given (F (2, 17) = 36.45, *p* < 0.001, η^2^ = 0.29), (b) the total number of rule violations (F (2, 17) = 113.09, *p* < 0.001, η^2^ = 0.56), and (c) the total achievement score (F (2, 17) = 47.75, *p* < 0.001, η^2^ = 0.35).

**Figure 2:**
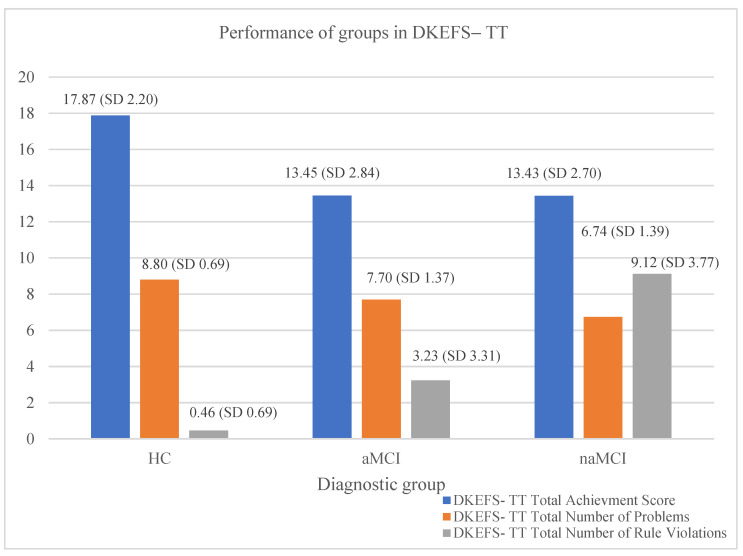
The effects of diagnostic group on the three scores of the DKEFS– Tower Test.

Scheffe’s post hoc comparisons revealed that HC older adults solved more problems compared to both aMCI I-J = 1.40, *p* = 0.001 and naMCI groups I-J = 2.06, *p* = 0.001; the performance of aMCI compared to naMCI was not significantly different (see Figure 2).

Μore interestingly, significant differences were found in the total number of rule violations across all three groups. The HC group committed fewer violations of the rules compared to the aMCI group, I-J = −2.77, *p* = 0.001, and the naMCI group, I-J = −8.66, *p* = 0.001. Significant differences were also observed between the two subtypes of MCI, with aMCI having fewer rule violations than naMCI, I-J = −5.89, *p* = 0.001 (see Figure 2).

Regarding the total achievement score, significant differences were observed among the groups as well, with the HC group showing a higher score compared to the aMCI group, I-J = 4.42, *p* = 0.001, and the naMCI group, I-J = 4.44, *p* = 0.001. However, the aMCI group and the naMCI group were not found to have a statistically significant difference in their performance regarding the total achievement score (see Figure 2).

Statistically significant differences were not observed in the performance of the three groups in the D-KEFS VF and D-KEFS C-WIT tests.

### 3.2. Subjective Sleep Measures: Group Differences in AIS, Stop-Bang, and PSQI Diary

The total scores for each participant on the AIS, Stop-Bang, and PSQI tests were calculated. We used MANOVA to explore the differences in sleep measures between the three groups on the above questionnaires. As dependent variables, the three total scores for the AIS, Stop-Bang, and PSQI tests were identified, and the diagnostic group was identified as the independent variable. No statistically significant differences in the mean values of the AIS, PSQI, and Stop-Bang tests were found between the three groups (see Table 2).

In evaluating subjective sleep disturbances, the AIS scores revealed a trend towards higher insomnia symptoms in individuals with aMCI compared to the HCs and those with naMCI. Conversely, the Stop-Bang scores were relatively similar across all groups, indicating a low to moderate risk for sleep apnea syndrome. Additionally, the PSQI scores did not show significant variation among the groups, indicating that overall subjective sleep quality was comparable. These results suggest that while insomnia symptoms may be more pronounced in the aMCI group, overall subjective sleep quality and risk of sleep apnea appear consistent across the different cognitive conditions.

The results from the sleep diary were ultimately not used due to a high number of incomplete or inaccurately filled entries.

### 3.3. Objective Sleep Measures: Group Differences in Actigraphy

**Figure 3:**
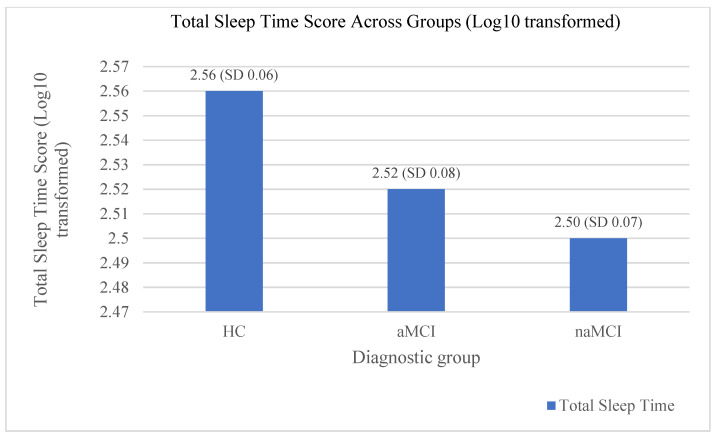
The effects of diagnostic group on total sleep time performance.

Initially, we transformed the data using a logarithmic scale (log10). The data from the actigraphy for each participant on total sleep time (TST), sleep efficiency (SE), and wakefulness after sleep onset (WASO) were calculated. MANOVA was conducted to examine the differences in objective sleep measures between the three groups. The results indicated a statistically significant effect of the diagnostic group on the actigraphy variables, V = 0.096, F (6.350) = 2.951, *p* < 0.008, η^2^ = 0.05. However, the diagnostic group effect was significant only for the variable TST, F (2.176) = 6.747, *p* = 0.002, η^2^ = 0.07. Scheffe’s multiple comparisons showed that the HC group had a longer TST compared to the naMCI group, I-J = 0.056, *p* = 0.002. Also, the results showed that HCs tended to differ in their performance with aMCI, I-J = 0.35, *p* = 0.055. MCI subtypes did not differ statistically significantly from each other (see Figure 3).

Notably, regardless of the log10 transformation, the average sleep durations in hours for the three groups were as follows: 6.13 for the HC, 5.72 for the aMCI, and 5.44 for the naMCI group.

The results indicate that there is no statistically significant difference between the three groups regarding sleep efficiency and wakefulness after sleep onset (see Table 3).

Specifically, the mean sleep efficiency was comparable among all groups, and WASO values were similarly low across conditions. These findings suggest that within this sample, sleep quality and fragmentation may not differ significantly between the cognitive impairment groups and the healthy controls.

### 3.4. Correlations between All Variables of Interest

Pearson correlation coefficients were used to analyze the relationships between the variable total sleep time and each variable of D-KEFS–TT, SF (total number of problems given, total number of violations, and total achievement score) (Table 4). Three significant correlations were observed between total sleep duration and performance on D-KEFS–TT, SF only for the total sample.

### 3.5. Mediation Analyses

In the analyses that followed, diagnosis was defined as the predictor, D-KEFS–TT performances were defined as the outcome variables, and total sleep time was the mediator. Separate mediation analyses were performed for (a) the aMCI and healthy control groups, (b) the naMCI and healthy control groups, and (c) the aMCI and naMCI groups. No significant indirect effects were found. Hence, the significant correlations of the variables of interest could represent the potential common detrimental effects of other (possibly biological) ‘causes’ related to MCI pathology.

## 4. Discussion

Τhe present study examined the relationships between specific cognitive control abilities and objective and subjective sleep parameters in individuals with aMCI, naMCI, and healthy controls. The analyses revealed statistically significant differences between groups in cognitive planning, with healthy people performing better than both aMCI and naMCI in terms of rule violations and total achievement score in the Tower Test. Actigraphy showed significant differences only in sleep duration, with healthy people having a longer total sleep time compared to the naMCI group, as well as a tendency to outperform aMCI. Correlations between total sleep time and cognitive planning performances showed significant relationships for the entire sample, without identifying significant indirect effects of diagnosis, via sleep duration, on cognitive planning. Generally speaking, it is of great interest that both MCI subtypes’ diagnosis is not associated with generalized cognitive control problems but only with complex, combinatory control, and also with generalized sleep disturbances except for a specific decrease in sleep duration. The results contribute to the increasing understanding of cognition and sleep in MCI. Below, we will discuss the findings in light of the study’s objectives and hypotheses, consider the implications, and propose directions for future research.

### 4.1. Cognitive Control Differences among Diagnostic Groups

Individuals with MCI often exhibit selective impairments in complex cognitive functions, such as cognitive planning, rather than a uniform decline across all cognitive domains. Cognitive planning refers to the array of neuropsychological processes involved in structuring, assessing, and selecting a series of thoughts and actions to attain specific objectives. It is regarded as a combinatory, complex executive function skill, incorporating elements such as the ‘updating’ aspect of working memory, inhibitory control, and task/rule switching [33]. The pattern of the findings suggests a decline in cognitive planning abilities from healthy aging to MCI, beginning with a clear inability to produce and maintain rules effectively. Rule violations are a type of error that has shown clinical utility and anatomical specificity.

Studies have demonstrated that deficits in MCI are especially pronounced in tasks requiring intricate and intact cognitive functioning [46,47]. This selective impairment could be attributed to the relatively early stage of the condition, typically diagnosed within 1–2 years of onset, where the cognitive decline has not yet generalized across all executive functions [48]. Thus, the pattern of impairment reflects the disease’s developmental phase and its impact on complex cognitive tasks. Moreover, the two MCI groups were found to significantly differ in only one planning aspect, that of rule violations, where naMCI performed significantly lower than aMCI. Rule violations can be categorized into two levels—(i) simple rule violations, where basic adherence to established rules is assessed, and (ii) complex rule violations, which involve a more nuanced understanding and application of the rules in varying contexts [49].

Our findings indicate that naMCI individuals performed significantly worse in complex rule violations compared to aMCI individuals. This disparity may reflect underlying differences in cognitive flexibility and executive control. Research has suggested that naMCI is characterized by more pronounced impairments in adaptive rule application and cognitive flexibility, which are crucial for managing complex rules [50]. In contrast, aMCI tends to present with less severe disruptions in these processes, possibly due to the nature and progression of cognitive decline in aMCI being less advanced or differently structured [51].

According to recent studies, individuals with naMCI exhibit greater deficits in cognitive planning tasks compared to those with aMCI, primarily due to distinct neuropathological mechanisms. NaMCI is closely associated with atrophy and functional impairments in the prefrontal cortex (PFC), particularly the dorsolateral prefrontal cortex (DLPFC), which is crucial for executive functions like planning and decision-making. Additionally, disruptions in the frontoparietal and cingulo-opercular networks, which involve the PFC and anterior cingulate cortex (ACC), further impair cognitive control. These networks are essential for maintaining attention and managing working memory, and their disruption leads to difficulties at least in complex executive functioning tasks that require combinations of abilities [52,53].

Moreover, naMCI is frequently associated with a greater extent of cerebrovascular pathology, including white matter hyperintensities and microinfarcts, which negatively impact the fronto-subcortical circuits responsible for executive functions [54]. This condition is also associated with a wider range of neuropathologies, such as Lewy body disease and frontotemporal lobar degeneration, which mainly affect the frontal and frontostriatal circuits. Conversely, aMCI is generally linked to atrophy in the medial temporal lobe and Alzheimer’s disease pathology, which primarily impacts memory rather than executive functions. Consequently, individuals with naMCI, who have lower neural reserves in the prefrontal regions, demonstrate a diminished ability to compensate for cognitive deficits, leading to more significant impairments in cognitive planning tasks [55]. These observations underscore the need to distinguish between aMCI and naMCI in order to develop targeted interventions for each subtype.

This specific focus on rule violations in cognitive planning is noteworthy, as it highlights a targeted aspect of executive functioning that is affected differently across MCI subtypes. Although this finding was not hypothesized in the introduction, it is significant because it underscores the nuanced impact of MCI on executive functions, revealing a very specific impairment pattern that has not been previously documented.

### 4.2. Sleep Disturbances and MCI

In evaluating subjective and objective sleep measures across different cognitive impairment groups, our study found that while sleep efficiency and WASO did not differ significantly, TST was notably reduced in participants with MCI, particularly in the naMCI group. These findings align with the broader literature, which suggests that while sleep disturbances are commonly associated with neurodegenerative conditions like Alzheimer’s disease, the link between MCI and sleep disturbances remains less clear.

Despite the absence of significant age differences between the groups, it is important to consider that age could potentially influence sleep metrics, particularly in older populations. While the age similarity suggests that it may not have had a major impact on the overall results, age-related factors should still be considered in future studies. Additionally, the Stop-Bang scores indicate a low to moderate risk of obstructive sleep apnea syndrome (OSAS) across all groups, which could also affect sleep quality. Despite the lack of significant differences in the Stop-Bang scores between the groups, the prevalence of OSAS in this age group remains a relevant factor to consider when interpreting the sleep data.

It is very interesting to note that not all studies have found a clear association between MCI and sleep disturbances. This is true for the present study too. Some studies suggest that while there may be a correlation between certain types of dementia (such as Alzheimer’s disease) and sleep disturbances, the relationship between MCI and sleep remains less clear [56,57,58]. Thus, Hypothesis 1 is only partially confirmed, in terms of the specific decrease in sleep duration that was found for our sample.

The differences in sleep reduction mechanisms between aMCI and naMCI reflect their underlying pathophysiological distinctions. Specifically, aMCI, associated with memory impairment, shows pronounced amyloid beta accumulation and tau pathology in brain regions like the hypothalamus and thalamus, which are crucial for sleep regulation. Neurofibrillary tangles and neuronal loss in the hippocampus, coupled with chronic inflammation and synaptic dysfunction, further disrupt sleep architecture, leading to a reduced total sleep time [59,60].

Conversely, naMCI is associated with impairments in cognitive areas beyond memory, such as executive function and visuospatial abilities. This subtype is linked to different neuroanatomical pathways, neurotransmitter imbalances, and a higher burden of white matter lesions, often due to vascular contributions to cognitive impairment. These factors disrupt communication between brain regions that are crucial for sleep regulation.

The integration of the Defensive Activation Theory and Active Inference Theory into this context provides additional insights into the role of sleep in MCI. According to the Defensive Activation Theory, REM sleep is crucial for processing emotional experiences and managing stress, which can be disrupted in both MCI subtypes, exacerbating emotional dysregulation [61,62]. The Active Inference Theory further suggests that sleep, especially REM sleep, helps in consolidating memories and updating predictive models, a process that may be compromised in aMCI due to underlying neurodegenerative processes [62]. These theories suggest that disturbances in REM sleep could contribute to cognitive decline in each of the subtypes of MCI by impairing emotional regulation or/and memory consolidation [61].

For instance, Eagleman and Vaughn (2021) proposed that REM sleep functions as a mechanism to prevent the visual cortex from being overtaken by non-visual processes, highlighting the potential consequences of REM sleep disruption in neurological conditions like idiopathic REM sleep behavior disorder (iRBD) [63]. Furthermore, recent perspectives by Antonioni et al. (2024) suggest that positive neurological symptoms might not only be expressions of damage, but could also represent self-defense mechanisms of the brain, as outlined by the Defensive Activation Theory [64]. These insights underscore the importance of these theoretical frameworks in explaining the complex interplay between sleep and neurological disorders, particularly in the context of MCI.

Together, these distinct mechanisms underscore the complex interplay between neurodegeneration, vascular changes, and neurotransmitter alterations in MCI subtypes. Hence, all these alterations seem to result in lowering the hours of sleep in MCI, as compared to healthy cognition in aging. However, sleep duration appears to be the only sleep parameter that changes in MCI, and the question raised is whether this parameter is enough to contribute to the decline in cognitive planning in MCI.

According to the extant literature, a short sleep duration may reduce cognition due to insufficient sleep, potentially promoting neurodegeneration by causing neuroinflammation and disrupting neurogenesis [65]. MCI is also associated with higher cortisol levels, which are related to shorter sleep duration and greater cognitive deterioration [66].

Thus, sleep duration could serve as an early marker for the risk of future cognitive decline and potentially act as a modifiable risk factor during midlife, helping to reduce the rising incidence of dementia cases.

### 4.3. Connection between Sleep Duration and Cognitive Planning

Research has delved into various biological mechanisms that might explain the connection between sleep and executive functions. These mechanisms encompass possible shifts in brain volume, changes in brain connectivity, the buildup of neurodegenerative proteins, and disturbances in glymphatic drainage [67,68]. During sleep, the brain undergoes various processes that are essential for memory consolidation, synaptic plasticity, and overall cognitive recovery [69,70]. Disruptions in sleep patterns, such as insufficient sleep duration, can impair these processes, leading to deficits in complex cognition [71].

As anticipated, in the present study, associations were identified only between cognitive planning and total sleep time, as regards the total sample, partially confirming Hypothesis 2a. Moreover, the findings from the mediation analysis showed that there is not any effect of sleep duration on the three variables of planning. Thus, a common underlying mechanism may explain the associations between sleep duration and cognitive planning changes as a potential cause of them.

The positive correlations of sleep duration with total achievement score could suggest that a longer sleep duration is linked to an enhanced ability to recognize and solve problems in a long-term planning condition, and this seems to be true for both healthy controls and those with MCI during aging. Moreover, the negative correlation between total sleep time and the total number of rule violations indicates that people who sleep more tend to make fewer rule violations. Several biological mechanisms can explain and support these correlations. Firstly, it can be attributed to neuroplasticity and neurogenesis. Adequate sleep can enhance neuroplasticity in brain regions, by promoting synaptic growth and strengthening synaptic connections, and these benefits of adequate sleep can potentially support complex cognition such as cognitive planning. Hence, a shorter sleep duration can disrupt complex cognition via decreasing neuroplasticity in the brain [65,71,72,73].

Additionally, toxin removal is another biological process that appears to support the findings. The activity of the glymphatic system increases during sleep, clearing toxic proteins from the brain [49,74]. Effective toxin removal during sleep can slow potential neurodegeneration by reducing amyloid-β levels, thereby mitigating further cognitive decline regarding potential vascular changes and the presence of white matter lesions. Sleep facilitates the clearance of metabolic waste products and reduces neuroinflammation, which is crucial in preventing vascular damage [75]. By enhancing the removal of toxins, sleep can help maintain the integrity of white matter tracts, supporting better connectivity between brain regions involved in cognitive planning. This can lead to fewer cognitive disruptions and improved complex cognitive control [76]. Furthermore, the regulation of hormonal balance seems to explain the correlations to some extent. Sleep is crucial for regulating cortisol levels, a hormone associated with stress. High cortisol levels due to sleep deprivation can negatively affect at least the combinatory and more demanding executive functions [66,73,77].

## 5. Limitations

It is necessary to consider the limitations of the present study. Reliance on actigraphy without polysomnography (PSG) may limit sleep measurement accuracy, and the absence of structural MRI and biomarkers like amyloid-β and PET scans hinders linking sleep disturbances to biological changes in MCI and Alzheimer’s disease. However, actigraphy remains a valuable tool in objectively capturing sleep–wake patterns. The inclusion of early-stage MCI participants may reduce observable differences from healthy controls. Additionally, the cross-sectional design of the study does not allow for the tracking of changes in variables of interest over time, limiting the ability to observe trajectories of change. It should be noted that a longitudinal study has already been completed, which will provide further insights into these trajectories. These limitations highlight the need for future studies to expand our knowledge of specific sleep parameters’ effects on specific cognitive functions in MCI populations.

## 6. Conclusions

The findings have some significant implications for both basic and applied research. They support the view that adequate sleep duration is connected to cognitive planning, a form of complex cognition, in all older adults, both healthy controls and those with MCI. Based on these findings, it is suggested that improving sleep duration could be a crucial means to preserve long-term planning in real-life situations of older adults, even if the relationship between sleep duration and cognitive planning is not direct and causal. A series of biological mechanisms, such as synaptic plasticity, glymphatic system and waste clearance, neurotransmitter regulation, hormonal regulation and brain connectivity, and network functioning can explain the relationship.

## 7. Future Implications

This study underscores the need for longitudinal studies measuring sleep duration over extended periods, as well as valid sleep intervention studies, to elucidate the connection between habitual sleep patterns and cognition in aging, in both pathological and healthy individua;s. Additionally, investigating the interaction between sleep, circadian factors, and cognitive performance, as well as the impact of MCI on sleep parameters and cognitive functions, is crucial for advancing our understanding of the specific relationships among the variables of interest.

## Figures and Tables

**Table 1:** Participants’ demographic characteristics.

	HC	aMCI	naMCI
	Mean (SD)	Mean (SD)	Mean (SD)
Age	70.28 (5.02)	71.09 (4.98)	69.07 (4.18)
Education	11.98 (3.09)	12.41 (3.18)	12.57 (3.39)
Gender (f/m)	36/10	51/24	39/19

**Table 2:** Participants’ subjective sleep data.

	HC	aMCI	naMCI
	Mean (SD)	Mean (SD)	Mean (SD)
AIS Score	5.26 (3.58)	6.44 (4.53)	4.76 (4.47)
Stop-Bang Score	3.11 (1.10)	2.79 (1.11)	2.69 (1.30)
PSQI Score	5.65 (2.08)	5.60 (2.66)	4.74 (2.05)

**Table 3:** Participants’ objective sleep data.

	HC	aMCI	naMCI
	Mean (SD)	Mean (SD)	Mean (SD)
Sleep efficiency (SE)	77.61 (8.58)	77.76 (10.58)	76.74 (8.91)
Wake after sleep onset (WASO)	1.79 (0.35)	1.76 (0.31)	1.81 (0.29)

**Table 4:** Correlations between total sleep time and D-KEFS–Tower Test variables in the total sample.

	Total Achievement Score	Total Number of Problems	Total Number of Rule Violations
Total sleep time	0.158 *	0.169 *	−0.229 **

* *p* < 0.05. ** *p* < 0.01.

## Data Availability

The data are unavailable due to privacy reasons and participant’s ethical restrictions, since the study’s participants did not give informed consent to data sharing.
